# Supplementary material for: Zeta CrAss-like Phages, a Separate Phage Family Using a Variety of Adaptive Mechanisms to Persist in Their Hosts
Source: Int J Mol Sci. 2025 Aug 8;26(16):7694. doi: 10.3390/ijms26167694 (PMC12386605; doi:10.3390/ijms26167694)
Supplement: Supplementary file 1 [file ijms-26-07694-s001.zip › ijms-3784176-supplementary/Figure S1_Babkin_.pdf]

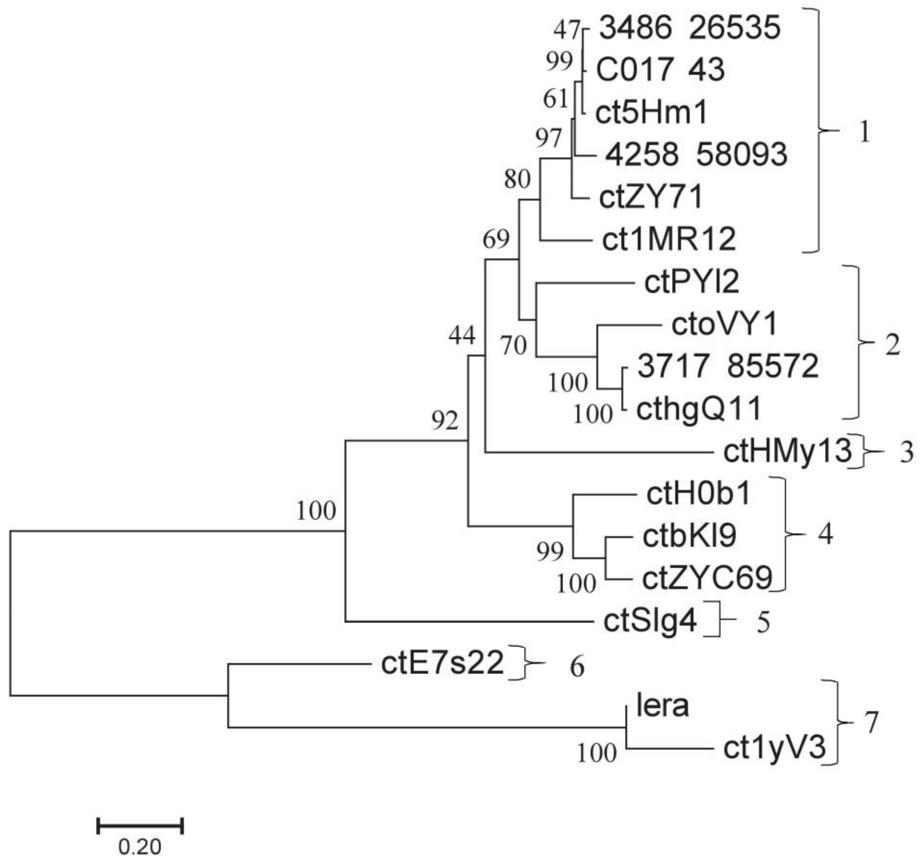

Figure S1. Maximum Likelihood phylogenetic tree of the TFPs encoded by the target genes in various DGRs of Zeta crass-like phages generated using IQ-tree software. Bootstrap values calculated from 500 replicates are given at the nodes. The scale bar represents the number of substitutions per site. The numbers of clades discussed in the text are shown at the right.
