# Supplementary material for: Zeta CrAss-like Phages, a Separate Phage Family Using a Variety of Adaptive Mechanisms to Persist in Their Hosts
Source: Int J Mol Sci. 2025 Aug 8;26(16):7694. doi: 10.3390/ijms26167694 (PMC12386605; doi:10.3390/ijms26167694)
Supplement: Supplementary file 1 [file ijms-26-07694-s001.zip › ijms-3784176-supplementary/Table S1_Babkin.pdf]

Table S1. Annotation of the Lera genome

#	Putative product	Coordinates, bp	Length, bp	Direction	Type	Amber suppession
1.	hypothetical protein	95..604	510	+	CDS	-
2.	hypothetical protein	828..1064	237	+	CDS	-
3.	hypothetical protein	1036..1263	228	+	CDS	+
4.	oriC region	1264..1624	361	+	region	-
5.	hypothetical protein	1625..1936	312	+	CDS	-
6.	methyltransferase	2146..2733	588	+	CDS	-
7.	hypothetical protein	2735..2878	144	+	CDS	-
8.	hypothetical protein	2866..3096	231	+	CDS	-
9.	hypothetical protein	3134..3376	243	+	CDS	-
10.	hypothetical protein	3380..3571	192	+	CDS	-
11.	hypothetical protein	3578..3772	195	+	CDS	-
12.	hypothetical protein	3852..4292	441	+	CDS	-
13.	hypothetical protein	4313..5122	810	+	CDS	+
14.	hypothetical protein	5200..5925	726	+	CDS	-
15.	cas system-associated protein	5944..6105	162	+	CDS	-
16.	hypothetical protein	6107..6346	240	+	CDS	-
17.	hypothetical protein	6334..6738	405	+	CDS	-
18.	hypothetical protein	6740..7093	354	+	CDS	-
19.	hypothetical protein	7165..7518	354	+	CDS	-
20.	tRNA	7701..7771	71	+	tRNA	-
21.	tRNA	7948..8021	74	+	tRNA	-
22.	tRNA	8077..8161	85	+	tRNA	-
23.	outer membrane protein A	8201..9280	1080	+	CDS	-
24.	hypothetical protein	9358..9645	288	+	CDS	-
25.	tRNA	9827..9899	73	+	tRNA	-
26.	tRNA	10,053..10,124	72	+	tRNA	-
27.	HNH homing endonuclease	10,189..10,665	477	+	CDS	-
28.	tRNA	10,712..10,787	76	+	tRNA	-
29.	hypothetical protein	10,846..10,977	132	+	CDS	-
30.	putative transposase	11,021..11,257	237	+	CDS	-
31.	tRNA	11,395..11,482	88	+	tRNA	-
32.	HNH endonuclease	11,485..11,670	186	+	CDS	-
33.	hypothetical protein	11,672..11,941	270	+	CDS	-
34.	hypothetical protein	11,941..12,135	195	+	CDS	-
35.	hypothetical protein	12,137..12,775	639	+	CDS	+
36.	hypothetical protein	12,748..13,608	861	+	CDS	-
37.	hypothetical protein	13,652..13,813	162	+	CDS	+
38.	tRNA	13,853..13,925	73	+	tRNA	-
39.	tRNA	14,128..14,199	72	+	tRNA	-
40.	tRNA	14,242..14,315	74	+	tRNA	-
41.	tRNA	14,322..14,396	75	+	tRNA	-
42.	hypothetical protein	14,478..14,618	141	+	CDS	-
43.	hypothetical protein	14,699..14,926	228	+	CDS	-
44.	tRNA	14,934..15,008	75	+	tRNA	-
45.	exodeoxyribonucleas	15,029..15,367	339	+	CDS	-
46.	tRNA	15,505..15,579	75	+	tRNA	-
47.	hypothetical protein	15,610..16,119	510	+	CDS	-
48.	tRNA	16,149..16,221	73	+	tRNA	-
49.	tRNA	16,584..16,675	92	+	tRNA	-

50.	hypothetical protein	16,692..16,958	267	+	CDS	-
51.	tRNA	16,963..17,034	72	+	tRNA	-
52.	tRNA	17,048..17,119	72	+	tRNA	-
53.	tRNA	17,136..17,207	72	+	tRNA	-
54.	tRNA	17,216..17,300	85	+	tRNA	-
55.	tRNA	17,694..17,787	94	+	tRNA	-
56.	hypothetical protein	18,504..18,647	144	+	CDS	-
57.	phosphoribosyltransferase	18,659..19,192	534	+	CDS	-
58.	tRNA	19,204..19,277	74	+	tRNA	-
59.	putative structural protein	19,394..20,119	726	+	CDS	-
60.	MazG nucleotide pyrophosphohydrolase	20,122..20,427	306	+	CDS	-
61.	hypothetical protein	20,427..20,837	411	+	CDS	-
62.	DUF6383 domain-containing protein	20,839..22,155	1317	+	CDS	-
63.	hypothetical protein	22,184..22,333	150	+	CDS	-
64.	hypothetical protein	22,335..22,577	243	+	CDS	-
65.	hypothetical protein	22,570..23,292	723	+	CDS	+
66.	ribonuclease HI	23,361..23,624	264	+	CDS	-
67.	hypothetical protein	23,635..23,748	114	+	CDS	-
68.	acetyltransferase	23,767..24,864	1098	+	CDS	-
69.	DUF2135 domain-containing protein	24,880..26,886	2007	+	CDS	-
70.	tail assembly chaperone	26,899..27,282	384	+	CDS	-
71.	hypothetical protein	27,295..27,654	360	+	CDS	-
72.	lipoprotein	27,636..27,893	258	+	CDS	-
73.	N-acetylmuramoyl-L-alanine amidase	27,833..28,264	432	+	CDS	-
74.	hypothetical protein	28,254..28,412	159	+	CDS	-
75.	membrane protein	28,443..29,648	1206	+	CDS	-
76.	tRNA	29,762..29,834	73	+	tRNA	-
77.	hypothetical protein	29,859..30,239	381	+	CDS	-
78.	putative transcription regulation protein	30,223..30,351	129	+	CDS	-
79.	cell division protein	30,436..30,732	297	+	CDS	-
80.	replication restart DNA helicase	30,704..30,907	204	+	CDS	-
81.	hypothetical protein	30,856..31,179	324	+	CDS	+
82.	hypothetical protein	31,122..31,331	210	+	CDS	-
83.	hypothetical protein	31,270..31,500	231	+	CDS	-
84.	ferritin	31,503..32,177	675	+	CDS	-
85.	hypothetical protein	32,200..32,697	498	+	CDS	-
86.	cell division protein	32,698..33,096	399	+	CDS	-
87.	hypothetical protein	33,059..33,370	312	+	CDS	-
88.	hypothetical protein	33,372..33,638	267	+	CDS	-
89.	hypothetical protein	33,626..33,799	174	+	CDS	-
90.	hypothetical protein	33,799..34,077	279	+	CDS	-
91.	hypothetical protein	34,077..34,397	321	+	CDS	-
92.	hypothetical protein	34,397..34,717	321	+	CDS	-
93.	hypothetical protein	34,788..35,648	861	+	CDS	-
94.	DNA repair helicase	35,756..37,018	1263	+	CDS	-
95.	hypothetical protein	37,066..37,293	228	+	CDS	-
96.	hypothetical protein	37,287..37,457	171	+	CDS	-

97.	hypothetical protein	37,464..37,682	219	+	CDS	+
98.	DnaD domain-containing protein	37,727..38,305	579	+	CDS	-
99.	replicative DNA helicase	38,305..39,417	1113	+	CDS	-
100.	AAA family ATPas	39,417..40,166	750	+	CDS	-
101.	hypothetical protein	40,166..40,750	585	+	CDS	-
102.	single-stranded DNA-binding protein	40,837..41,547	711	+	CDS	-
103.	DNA primase	41,592..42,596	1005	+	CDS	-
104.	holliday junction resolvase	42,589..43,140	552	+	CDS	-
105.	hypothetical protein	43,163..43,426	264	+	CDS	-
106.	ADP-ribosylglycohydrolase	43,427..44,245	819	+	CDS	-
107.	hypothetical protein	44,239..44,451	213	+	CDS	-
108.	5' nucleotidase	44,452..45,075	624	+	CDS	+
109.	exodeoxyribonuclease	45,165..46,193	1029	+	CDS	-
110.	hypothetical protein	46,183..46,560	378	+	CDS	-
111.	hypothetical protein	46,807..47,361	555	+	CDS	-
112.	hypothetical protein	47,493..49,196	1704	+	CDS	-
113.	hypothetical protein	49,099..49,521	423	+	CDS	-
114.	hypothetical protein	49,530..50,306	777	+	CDS	-
115.	ribonucleoside-triphosphate reductase	50,378..52,804	2427	+	CDS	-
116.	UvrD-like helicase	52,806..54,284	1479	+	CDS	-
117.	hypothetical protein	54,341..54,490	150	+	CDS	-
118.	hypothetical protein	54,519..54,665	147	+	CDS	-
119.	phosphoesterase	54,675..55,391	717	+	CDS	-
120.	outer membrane protein assembly factor BamE	55,384..55,635	252	+	CDS	-
121.	DUTPase	55,637..56,179	543	+	CDS	-
122.	hypothetical protein	56,179..56,658	480	+	CDS	+
123.	hypothetical protein	56,595..56,843	249	+	CDS	-
124.	DNA ligase	56,825..58,030	1206	+	CDS	-
125.	hypothetical protein	58,023..58,208	186	+	CDS	-
126.	hypothetical protein	58,208..58,468	261	+	CDS	-
127.	hypothetical protein	58,458..59,129	672	+	CDS	-
128.	RNA ligase	59,144..60,505	1362	+	CDS	-
129.	DUF4298 domain-containing protein	60,531..60,728	198	+	CDS	-
130.	hypothetical protein	60,728..61,435	708	+	CDS	-
131.	CCA tRNA nucleotidyltransferase	61,405..62,148	744	+	CDS	-
132.	hypothetical protein	62,151..62,375	225	+	CDS	-
133.	hypothetical protein	62,353..62,703	351	+	CDS	-
134.	hypothetical protein	62,703..62,948	246	+	CDS	-
135.	hypothetical protein	62,939..63,415	477	+	CDS	-
136.	Polynucleotide kinase	63,427..64,362	936	+	CDS	-
137.	hypothetical protein	64,385..64,558	174	+	CDS	-
138.	hypothetical protein	64,564..65,589	1026	+	CDS	-
139.	hypothetical protein	65,589..65,771	183	+	CDS	-
140.	ROK family protein	65,764..66,123	360	+	CDS	-
141.	UPF0346 protein	66,116..66,406	291	+	CDS	-
142.	hypothetical protein	66,406..66,585	180	+	CDS	-

143.	DNA polymerase I	66,594..68,783	2190	+	CDS	-
144.	hypothetical protein	68,843..69,187	345	+	CDS	-
145.	hypothetical protein	69,180..69,635	456	+	CDS	-
146.	thymidylate synthase	69,622..70,122	501	+	CDS	-
147.	Homing endonuclease	70,293..70,907	615	+	CDS	-
148.	hypothetical protein	70,923..71,096	174	+	CDS	-
149.	thymidylate synthase	71,105..71,470	366	+	CDS	-
150.	hypothetical protein	71,474..71,995	522	+	CDS	-
151.	putative phosphoesterase	72,008..72,334	327	+	CDS	-
152.	hypothetical protein	72,467..72,640	174	+	CDS	-
153.	hypothetical protein	72,621..72,824	204	+	CDS	-
154.	hypothetical protein	72,827..73,000	174	+	CDS	-
155.	SEC-C motif-containing protein	73,001..73,237	237	+	CDS	-
156.	phosphohydrolase	73,239..73,829	591	+	CDS	-
157.	hypothetical protein	73,816..74,094	279	+	CDS	-
158.	thoeris anti-defense protein	74,107..74,508	402	+	CDS	-
159.	hypothetical protein	74,509..74,760	252	+	CDS	+
160.	hypothetical protein	74,675..75,286	612	+	CDS	-
161.	DUF4157 domain-containing protein	75,279..75,665	387	+	CDS	-
162.	calcineurin-like phosphoesterase	75,646..76,362	717	+	CDS	-
163.	hypothetical protein	76,364..76,567	204	+	CDS	+
164.	nicotinamidase	76,485..77,066	582	+	CDS	-
165.	hypothetical protein	77,045..77,233	189	+	CDS	-
166.	nicotinate phosphoribosyltransferase	77,237..78,409	1173	+	CDS	-
167.	hypothetical protein	78,410..78,658	249	+	CDS	-
168.	glutamine-dependent NAD+ synthetase	78,666..79,571	906	+	CDS	-
169.	thymidylate kinase	79,594..80,112	519	+	CDS	-
170.	NMNAT protein	80,103..80,729	627	+	CDS	-
171.	MutT/nudix family protein	80,710..81,219	510	+	CDS	-
172.	signal peptidase	81,219..81,530	312	+	CDS	-
173.	hypothetical protein	81,518..81,700	183	+	CDS	-
174.	hypothetical protein	81,694..81,996	303	+	CDS	-
175.	NAD-dependent protein deacetylase	81,984..82,664	681	+	CDS	-
176.	putative prohead protease domain protein	82,658..83,074	417	+	CDS	-
177.	hypothetical protein	83,065..83,448	384	+	CDS	-
178.	hypothetical protein	83,465..83,875	411	+	CDS	-
179.	nucleotide-binding protein	83,878..84,654	777	+	CDS	-
180.	acid phosphatase	84,658..85,188	531	+	CDS	-
181.	aspartyl/glutamyl-tRNA(Asn/Gln) amidotransferase	85,178..85,573	396	+	CDS	-
182.	hypothetical protein	85,575..85,739	165	+	CDS	-
183.	hypothetical protein	85,727..85,852	126	+	CDS	-
184.	deoxynucleoside monophosphate kinase	85,891..86,436	546	+	CDS	-
185.	hypothetical protein	86,436..86,612	177	+	CDS	-

186.	exonuclease V	86,744..91,357	4614	-	CDS	+
187.	hypothetical protein	91,351..94,227	2877	-	CDS	+
188.	DNA-dependent RNA polymerase	94,227..96,857	2631	-	CDS	+
189.	homing endonuclease	97,289..97,981	693	-	CDS	-
190.	hypothetical protein	98,283..104,603	6321	-	CDS	-
191.	RecD-like protein	104,596..112,941	8346	-	CDS	+
192.	hypothetical protein	112,947..114,833	1887	-	CDS	-
193.	hypothetical protein	114,833..117,628	2796	-	CDS	+
194.	hypothetical protein	117,668..117,991	324	-	CDS	-
195.	DUF192 domain-containing protein	118,113..118,682	570	-	CDS	+
196.	hypothetical protein	118,682..120,235	1554	-	CDS	+
197.	muzzle protein	120,228..124,802	4575	-	CDS	+
198.	hypothetical protein	124,806..128,120	3315	-	CDS	-
199.	hypothetical protein	128,127..129,128	1002	-	CDS	+
200.	ring protein 1	129,196..129,933	738	-	CDS	+
201.	ring protein 2	129,933..130,643	711	-	CDS	-
202.	capsid protein	130,669..131,808	1140	-	CDS	-
203.	ring protein 3	131,821..132,750	930	-	CDS	+
204.	hypothetical protein	132,758..133,600	843	-	CDS	-
205.	major capsid protein	133,679..135,091	1413	-	CDS	-
206.	regulatory protein	135,115..136,203	1089	-	CDS	+
207.	RNA-binding protein	136,321..136,653	333	-	CDS	-
208.	tail protein	136,657..139,470	2814	-	CDS	+
209.	DUF5675 domain-containing protein	139,473..139,910	438	-	CDS	+
210.	hypothetical protein	140,122..140,436	315	-	CDS	-
211.	hypothetical protein	140,452..140,889	438	-	CDS	-
212.	exosporium-like protein	140,891..141,340	450	-	CDS	-
213.	tail protein	141,362..142,165	804	-	CDS	-
214.	chitin-binding type-3 domain-containing protein	142,428..143,426	999	-	CDS	-
215.	reverse transcriptase	143,429..144,628	1200	-	CDS	+
216.	TR1	144,654..144,702	49	-	region	-
217.	TR2	144,992..145,066	75	-	region	-
218.	putative TR3	145,078..145,129	52	-	region	-
219.	avd	145,251..145,604	354	-	CDS	-
220.	tail fiber protein	145,676..147,814	2139	-	CDS	+
221.	VR1	145,685..145,733	49	-	region	-
222.	VR2	145,726..145,800	75	-	region	-
223.	putative VR3	145,801..145,852	52	-	region	-
224.	tetracycline resistance protein	147,861..148,055	195	-	CDS	-
225.	baseplate J like protein	148,048..148,707	660	-	CDS	-
226.	baseplate J like protein	148,709..152,143	3435	-	CDS	+
227.	tail protein	152,147..153,583	1437	-	CDS	+
228.	hypothetical protein	153,588..153,932	345	-	CDS	+
229.	tail protein	153,977..156,769	2793	-	CDS	-
230.	tail protein	156,783..157,760	978	-	CDS	+
231.	tail protein	157,779..160,259	2481	-	CDS	+
232.	hypothetical protein	160,252..160,431	180	-	CDS	-
233.	hypothetical protein	160,415..161,143	729	-	CDS	+

234.	portal protein	161,145..163,331	2187	-	CDS	-
235.	terminase	163,560..165,887	2328	-	CDS	+
236.	terminase small subunit	165,890..166,468	579	-	CDS	-
237.	putative M15 family peptidase	166,492..167,547	1056	-	CDS	+
238.	BAH domain-containing protein	167,603..167,845	243	-	CDS	-
239.	chaperonin	167,852..168,406	555	-	CDS	-
240.	tRNA pseudouridine synthase	168,426..168,647	222	-	CDS	-
241.	DUF2769 domain-containing protein	168,617..168,856	240	-	CDS	+
242.	exonuclease V	168,976..169,878	903	-	CDS	-
243.	hypothetical protein	169,880..170,560	681	-	CDS	-
244.	cargo protein 1	170,720..171,028	309	+	CDS	-
245.	endolysin	171,030..173,117	2088	+	CDS	+
246.	holin	173,163..173,426	264	+	CDS	+
247.	hypothetical protein	173,392..174,204	813	+	CDS	-
248.	transglycosylase SLT domain-containing protein	174,204..174,989	786	+	CDS	+
249.	hypothetical protein	175,007..175,204	198	-	CDS	-
250.	ferredoxin/thioredoxin reductase	175,206..175,409	204	-	CDS	-
251.	hypothetical protein	175,387..175,623	237	-	CDS	-
252.	hypothetical protein	175,598..176,116	519	-	CDS	-
253.	tRNA	176,143..176,228	86	-	tRNA	-
254.	hypothetical protein	176,262..176,405	144	-	CDS	-
255.	tRNA	176,426..176,497	72	-	tRNA	-
256.	aminoacyl-tRNA hydrolase	176,509..176,781	273	-	CDS	+
257.	hypothetical protein	176,768..177,103	336	-	CDS	-
258.	tRNA	177,232..177,303	72	-	tRNA	-
259.	tRNA	177,309..177,403	95	-	tRNA	-
260.	hypothetical protein	177,410..177,598	189	-	CDS	-
261.	hypothetical protein	177,715..177,945	231	-	CDS	-
262.	hypothetical protein	177,911..178,066	156	-	CDS	-
263.	hypothetical protein	178,029..178,220	192	-	CDS	-
264.	hypothetical protein	178,193..178,486	294	-	CDS	+
265.	HNH endonuclease	178,526..179,476	951	-	CDS	-
